# Definitive Treatment of Early-Stage Non-Small Cell Lung Cancer with Stereotactic Ablative Body Radiotherapy in a Community Cancer Center Setting

**DOI:** 10.3389/fonc.2015.00146

**Published:** 2015-06-30

**Authors:** Cory Heal, William Ding, John Lamond, Michael Wong, Rachelle Lanciano, Stacy Su, Jun Yang, Jing Feng, Stephen Arrigo, Deborah Markiewicz, Alexandra Hanlon, Luther Brady

**Affiliations:** ^1^Drexel University College of Medicine, Philadelphia, PA, USA; ^2^Philadelphia CyberKnife, Philadelphia, PA, USA; ^3^Crozer Keystone Healthcare System, Philadelphia, PA, USA; ^4^David Geffen School of Medicine at UCLA, Los Angeles, CA, USA; ^5^Fox Chase Cancer Center, Philadelphia, PA, USA; ^6^University of Pennsylvania, Philadelphia, PA, USA

**Keywords:** cyberknife, non-small cell lung cancer, stereotactic body radiotherapy, stereotactic ablative radiotherapy, radiation oncology, XSight, radiation toxicity, early-stage lung cancer

## Abstract

**Introduction:**

Stereotactic ablative body radiotherapy (SABR) provides a superior non-small cell lung cancer (NSCLC) treatment option when compared to conventional radiotherapy for patients deemed inoperable or refusing surgery. This study retrospectively analyzed the rates of tumor control and toxicity following SABR treatment (Cyberknife system) of primary early-stage NSCLC in a community setting.

**Methods:**

One hundred patients were treated between 2007 and 2011. Patients with T3-4 or N1-3 disease, metastasis, recurrent local disease, or a non-lung primary were excluded from analysis. All patients had biopsy proven disease. Staging included CT or fluorodeoxyglucose-positron emission tomography scan. Median dose was 54 Gy (45–60); 18 Gy (10–20) per fraction. Median planned target volume expansion was 8 mm (2–10). Median BED was 151.2. Tumors were tracked via Synchrony, X-Sight Lung, or X-Sight Spine. Patients were evaluated for local control, overall survival (OS), and toxicity. All local failures were determined by evaluating post treatment PET/CT.

**Results:**

With a median follow up of 27.5 months, the 1-, 2-, and 3-year local control rates were 100, 93.55, and 84.33%, respectively. Median survival was 2.29 years; actuarial 3-year survival was 37.20%. Grade-3 toxicity was observed in 2% of patients (pneumonia within 2 months of treatment, *n* = 1; chronic pneumonitis requiring hospital admission, *n* = 1). No patients demonstrated toxicity above Grade-3. Multivariate analysis did not show T-stage as an independent predictor of OS, though it did trend toward significance.

**Conclusion:**

In a community-center setting, definitive treatment of NSCLC with SABR for non-surgical candidates and those who choose to forego surgery result in excellent and comparable rates of local control and toxicity compared to published series from large academic centers.

## Introduction

Since the report of the initial experience from Indiana University regarding the use of stereotactic ablative body radiotherapy (SABR) for early-stage non-small cell lung cancer (NSCLC) (1), there has been an explosion of interest and utility of this type of treatment. This form of treatment gives patients who are otherwise inoperable a new option with results that are generally superior to conventional radiotherapy (2). Operable patients who refuse surgery now also have this treatment alternative available (3–5). While many reports have come from large academic institutions, experiences at community hospital based centers are lacking. The need for more data from these centers is underscored as rapidly increasing numbers of community based centers are using SABR for the treatment of NSCLC.

The primary purpose of this study is to retrospectively investigate the rates of tumor control and toxicities related to the use of SABR in the treatment of primary early-stage NSCLC in a community center setting. The secondary purpose is to investigate potential tumor control differences using different techniques of planning and treatment delivery.

## Materials and Methods

Between January 2007 and August 2011, 100 patients who underwent definitive SABR at the Philadelphia CyberKnife for a stage I–II NSCLC were retrospectively reviewed from our patient database after receiving institutional review board approval (CKHS 14-006). Patients with T3–4 or N1–3 diseases, metastasis, small cell histology, absence of biopsy, recurrent disease, or a non-lung primary were excluded from the analysis. Patients included those deemed: (a) inoperable – based on pulmonary function tests, i.e., forced expiratory volume in 1 s (FEV1) <50% predicted or diffusing capacity of lung for carbon monoxide (DLCO) of <50% predicted, comorbidities, and recommendations from a multidisciplinary tumor board that included participation from radiation oncology, thoracic surgery, and medical oncology, as well as (b) operable ones who refused surgery.

All patients had biopsy proven disease. Staging was done with CT scanning and fluorodeoxyglucose-positron emission tomography (FDG-PET). All mediastinal staging were based on FDG-PET results.

Patients were treated on the CyberKnife^®^ stereotactic radiation therapy system (Accuray, Sunnyvale, CA, USA). Tumor tracking was accomplished with one of three methods: (a) fiducial tracking, (b) X-Sight Lung, which tracks the tumor directly, and (c) X-Sight Spine, which tracks a nearby vertebral body. CT simulation was done with three scans: regular inspiratory breath hold CT, expiratory breath hold, and free breathing CT. The expiratory hold CT was used for dosimetry calculation purpose. Contours were made on the MulitPlan^®^ planning system. In case of fiducial and X-Sight Lung tracking, only the expiratory breath hold scan was contoured to define the gross tumor volume (GTV) with planned target volume (PTV) generated by an 5–8 mm expansion. Using X-Sight Spine, all three phases were contoured to define the internal target volume (ITV), and the PTV was generated using a 5 mm expansion. Fractionation was determined using a risk adapted approach depending on tumor size and location. In general, patients with a peripheral tumor were treated to a dose of 60 Gy in 3 fractions before heterogeneity was accounted for, and 54 Gy in 3 fractions once we started using the Monte Carlo advanced dosimetry algorithm. Patients with a central tumor received 50 Gy (10 Gy × 5 fractions or 12.5 Gy × 4 fractions). The dosimetry algorithm used was Ray Tracing from 2007 to 2011, and then Monte Carlo from June 2011.

The first follow-up visit was typically at 1 month post-treatment, then every 3–4 months for 1 year, and annually thereafter. Follow-up CT scans were performed at each visit. FDG-PET scans were repeated at the managing physician’s discretion especially in cases where a growing lesion on CT could not be differentiated from tumor growth or fibrosis. Treatment response measurements were adopted from RECIST v1.1 (http://imaging.cancer.gov/clinicaltrials/imaging). Toxicity was scored based on the CTCAE v4 guidelines (6).

Local control (LC) is defined as the absence local failure. Local failure is defined either as primary tumor failure (PTF), marginal failure (MF) (within 1 cm of the PTV), or involved lobe failure (ILF). Regional failure is defined as failure in the regional lymph nodes. Distant failure is defined as failure outside of the local and regional areas.

Kaplan–Meier methodology was used to estimate outcomes of survival and LC, with comparisons accomplished using the log–rank statistics. Cox proportional hazards modeling was used to assess univariate and multivariable predictors of outcome. Final multivariable models were the result of building a full model comprised of all variables demonstrating significance at the 0.20 level on univariate analysis, followed by sequential elimination of the least significant variable until only those remaining in the model demonstrate significance at the 0.10 level. Statistical significance is concluded on the basis of a two-tailed *p*-value of 0.05.

## Results

From January 2007 to August 2011, 100 patient records with a median follow up of 27.5 months (range: 2–77 months) were analyzed. The median age at treatment was 75 years. Tumors were classified as centrally (27%) or peripherally (73%) located. Patient characteristics are summarized in Table 1.

**Table 1:** **Patient Characteristics**.

Patient Characteristics	Number of Patients
**Median Age (years)**	75 (60–88)
**Gender**
Male	53
Female	47
**Location**
Central	27
Peripheral	73
**Specific Path**
Adenocarcinoma	33
Squamous Cell	40
Large	2
NSCLC-NOS	25
**Stage**
T1	63
T2	37
**Tumor Size Median (cm)**	2.6

**Figure 1:**
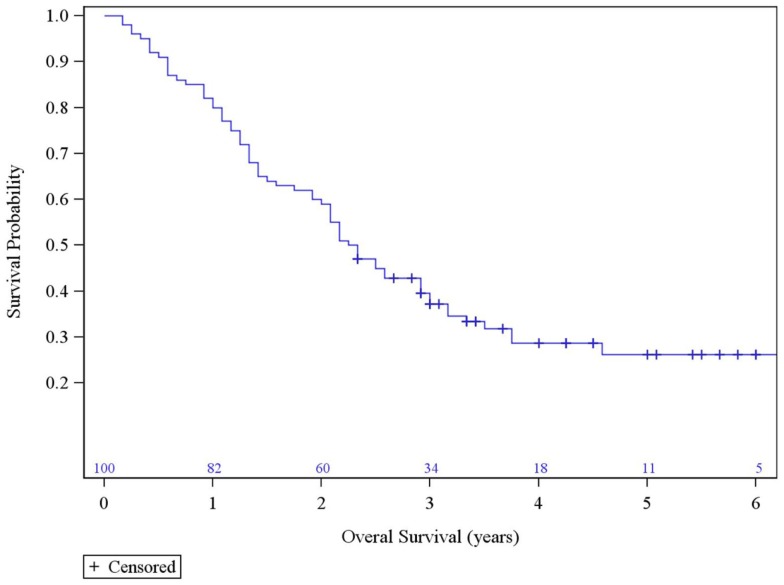
**Overall Survival**.

**Figure 2:**
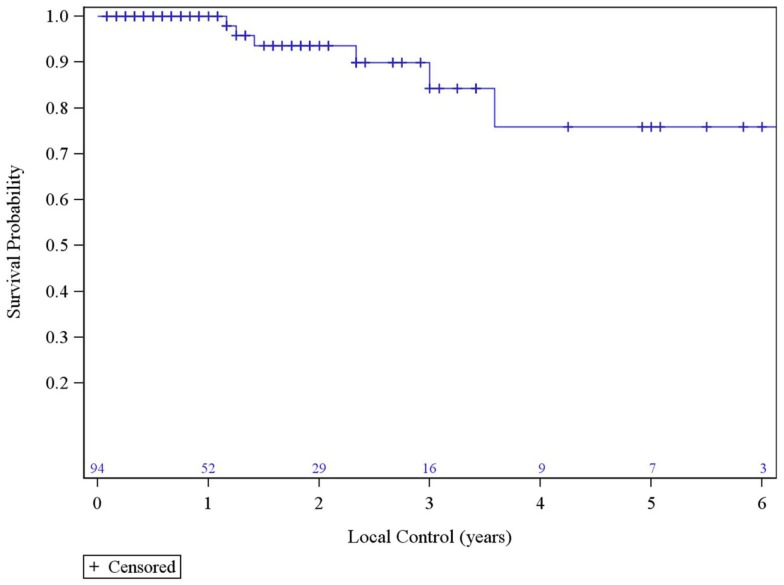
**Local Control**.

**Figure 3:**
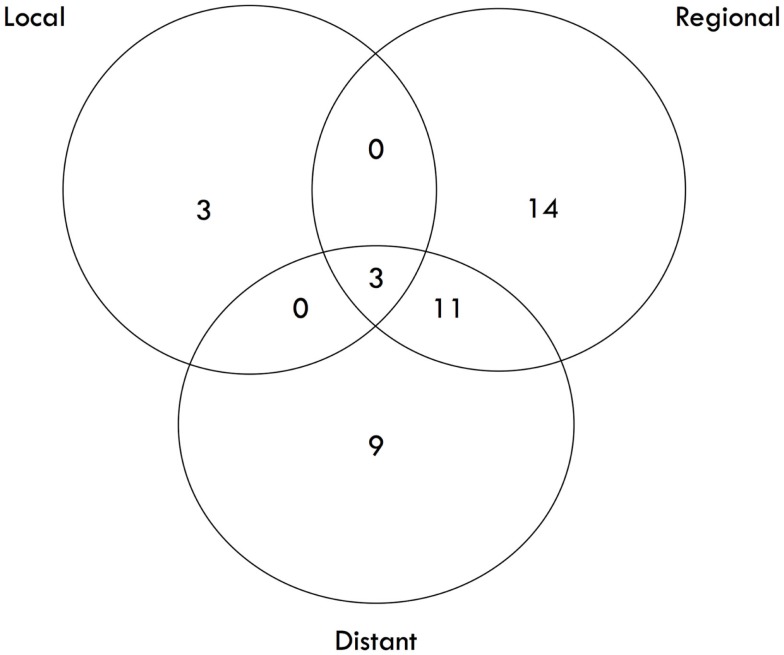
**Pattern of Failure**.

The median survival was 2.29 years and the 3-year overall survival (OS)was 37% (Figure 1). The Kaplan–Meier LC at 1-, 2-, and 3 years is 100, 94, and 84%, respectively (Figure 2). A total of 40 patients had cancer recurrence. The pattern of relapse included six local failures (4 PTF, 0 MF, and 2 ILF), 26 regional failures, and 20 distant failures. Of the T1 and T2 patients, 18 (28.6%) and 10 (27.0%) had regional failures, respectively. Distributions of the pattern of relapse are shown in Figure 3. The pattern of recurrence with 3 local only failures, 14 regional only failures, 9 distant only failures, 11 regional and distant failures, and 3 local, regional, and distant failures.

About 48% of patients were treated with fiducial tracking, 26% with X-sight Lung, and 26% with X-sight Spine. Of the six local failures, three were tracked using gold fiducials, two were tracked using X-Sight Spine, and one was tracked using X-Sight Lung. About 80% patients were planned with the Ray Tracking algorithm and 20% were with the Monte Carlo algorithm. No meaningful correlation of LC could be made between the two algorithms with only six local failures.

The median tumor size and PET SUV before treatment were 2.60 cm and 5.90 mg/mL, respectively. The most recent PET/CT of patients after treatment revealed a median tumor size and activity of 1.98 cm and 2.40 mg/mL, respectively. Other treatment characteristics are summarized in Table 2.

**Table 2:** **Treatment Characteristics**.

Treatment Characteristics	Median	Range	SD
BED dose (Gy_10_)	151	100–180	32.5
Prescription dose (Gy)	54	45–60	4.82
PTV margin (mm)	8	2–10	1.68
PTV volume (cm^3^)	34.4	8.3	117.9
Number of beams	107	42	207
Isodose line	70	60–84	5.4

The data from the univariate analysis are shown in Table 3. The resulting multivariate analysis showed neither T-stage nor BED as an independent predictor of OS (Table 4). However, T-stage did show a strong trend toward predictive value. No meaningful covariate analysis could be made with regard to LC due to the low number of events.

**Table 3:** **Univariate Analysis**.

Parameter	Frequency (%)	Hazard Ratio	95% Confidence Limit	*p*-Value
**T-Stage**				
T1	63	0.54	0.36–0.94	0.0267
T2	37	1.00		
**Gender**				
Female	53	1.05	0.66–1.67	0.8280
Male	47	1.00		
**Location**				
Central	27	0.74	0.43–1.28	0.2875
Peripheral	73	1.00		
**Histology**				
Adenocarcinoma	33	1.01	0.57–1.78	0.9707
Large cell	2	3.20	0.74–13.77	0.1183
NSCLC-NOS	25	0.94	0.51–7.50	0.8269
Squamous cell	40	1.00		
**Tumor Tracking**				
Fiducials	48	0.92	0.516–1.64	0.7771
X-Sight Lung	26	1.22	0.64–2.33	0.5474
X-Sight Spine	26	1.00		
**Dose Algorithm**				
Monte Carlo	20	1.53	0.83–2.74	0.1538
Ray Tracing	80	1.00		
**Plan Centricity**				
Isocentric	65	1.07	0.66–1.76	0.7817
Non-isocentric	35	1.00		
**Number of Fractions**				
3	62	2.50	0.98–6.32	0.0537
4	25	3.39	1.28–8.98	0.0139
5	13	1.00		
**BED Stratified**				
100–110 Gy	15	0.60	0.28–1.28	0.1836
111–120 Gy	25	1.26	0.74–2.15	0.4025
>120 Gy	60	1.00		
**PTV Margin**	–	0.94	–	0.3989
**Age**	–	1.03		0.0617

**Table 4:** **Multivariate analysis**.

Parameter	Hazard Ratio	95% Confidence Limits	*p*-Value
**T-Stage**			
T1	0.62	0.36–1.05	0.0737
T2	1.00		
**BED Stratified**			
100–110 Gy	0.50	0.22–1.12	0.0908
111–120 Gy	1.21	0.68–2.15	0.5114
>120 Gy	1.00		

Acute and chronic toxicities were evaluated in four categories: lung, esophagus, skin, and pain. Of 100 patients studied, two had toxicities scored at Grade 3 or above. They were Grade 3 toxicities for acute lung (1–90 days) due to acquiring pneumonia within 2 months of treatment (*n* = 1) and for chronic lung (>90 days) after acquiring chronic pneumonitis requiring hospital admission (*n* = 1). There were no acute or chronic Grade 3 toxicities for esophagus, skin, or pain, and no toxicities Grade 4 or above in any category (Table 5).

**Table 5:** **Acute and Chronic Toxicity Grading**.

	Grade 1–2	Grade 3	Grade 4–5
Acute lung	13	1^a^	0
Chronic lung	10	1^a^	0
Acute esophagus	4	0	0
Chronic esophagus	1	0	0
Acute skin	1	0	0
Chronic skin	0	0	0
Pain	9	0	0
Rib fracture	1	0	0

*^a^One patient with pneumonia within 2 months of treatment; one patient with chronic pneumonitis requiring hospitalization*.

## Discussion

There has been a rapid rise in the use of SABR for the definitive treatment of primary early-stage NSCLC for inoperable patients since the publication of the initial Indiana experience (7). Since then, more data have emerged that further substantiate the utility of this treatment method as an emerging standard of care for the inoperable patient population (8). There is, however, a paucity of published data from community-based cancer centers, which accounts for a significant part of this increase in utility.

To our knowledge, this is the largest series that has looked at this treatment modality in a community-based cancer center. Our results show a 3-year LC and OS that is in-line with the published series from large academic institutions (Table 6).

**Table 6:** **Comparable Publications**.

Author	*N*	Median F/U (months)	Median BED (Gy_10_)	3-year LC (%)	3-year OS (%)
Onishi et. al (9)	245	24	108	85	40
Baumann et. al (10)	138	33	112.5 (15 Gy × 3)	88	52
Timmerman et. al (11)	70	32	180 (151^a^)	88	42
Present Study	100	27	151	84	37

*^a^Heterogeneity correction equivalent*.

Compared to the pattern of relapse from the long-term update of RTOG 0236 (12), where the 5-year regional and distant progression are 38 and 31%, respectively, our results also demonstrated a large percentage of patients who experienced regional or distant failure (26 and 20%, respectively).

We reason that our reliance on PET as the primary staging method, while non-invasive, may underestimate the degree of regional lymph node involvement at the time of initial diagnosis, therefore giving way to increase in regional nodal failure. While mediastinoscopy staging is the gold standard, performing invasive mediastinal biopsies carries a risk to any patient, and may not even be possible for inoperable patients with significantly decreased pulmonary function. This dilemma highlights the potential utility of minimally invasive endobronchial ultrasound-guided transbronchial needle biopsy to evaluate hilar and mediastinal lymph nodes as a part of the staging work up (13).

With regards to the high rate of distant progression, this may be due to the presence of circulating tumor cells (CTC) that have already seeded or have the potential to seed locations outside the original tumor area (14–16). Even if a curative dose of radiation therapy is administered at the tumor site, other areas of the lung and organs are left untreated, which raises the important question of whether the number of CTC or the characteristics of these CTC (isolated vs. clustered) will predict for a greater role of adjuvant chemotherapy to prevent distant progression.

With regards to toxicity, our experience shows a favorable toxicity profile of having 2% Grade 3 toxicity, and no grade 4 or 5 toxicity. One reason for this may be due to our risk adaptive approach, as guided by other experiences (11, 17–19), in which central tumors and tumors close to other critical structures would receive a more fractionated regimen of 4–5 fractions, in an attempt to deliver a more tolerable dose to the normal tissue, but at the same time a potent enough dose of BED >100 Gy_10_ to the tumor (4, 20). Another reason could be due to technological improvements over time. Our ability to track the tumor throughout treatment in real-time with CyberKnife may improve accuracy of treatment, allowing for smaller PTV margins. This leads to less overall toxicity, while maintaining a comparable rate of LC. Others have also reported excellent toxicity data using real-time tracking (21).

Regarding covariate of treatment planning and delivery, neither algorithm or dose nor PTV margin was significant in predicting OS. During the study period, although the Ray Tracing algorithm was used 80% of the time, some of these plans were started with Ray Tracing but were then compared to a Monte Carlo estimate. This was done to leverage the efficiency of Ray Tracing, while keeping Monte Carlo as a gold standard. In general, Monte Carlo was used as a comparison for small tumors where there is inadequate dose build up due to tissue heterogeneity. If there was no significant difference between the estimates, then the Ray Tracing plan was executed. As of June 2011, all treatments were planned and executed using the Monte Carlo algorithm. Others have reported a dose–response relationship (17). While a dose–response relationship was not noted due to small number of local failures, we have demonstrated previously that Ray Tracing can significantly underdose small tumors by as much as 30–40% (22), and has been supported by others (23). We further postulate that even if there exists a dose–response relationship, that this difference may be too small to detect since all of our prescriptions have been given in a range above BED >100 Gy_10_ where there is evidence to suggest that a dose plateau may occur starting around 100 Gy BED (24, 25).

T-stage showed a strong trend toward being an independent prognostic factor for OS. This raises the hypothesis of whether using neoadjuvant chemotherapy to initially downstage the tumor before SABR, or using chemotherapy in the adjuvant setting will provide any additional benefit in patients with larger tumors. BED showed no independent predictive value related to OS. Again, this is likely due to the relatively high BED prescription (>100 Gy) and curative approach to treatment. It is interesting to note that a recent report found a survival benefit of using a prescription BED >150 Gy in patients with T2 tumors (26). In our study, the number of fractions was not included in the final multivariate model due to its high correlation with BED and the possibility of confounding the data.

Limitations of this study include the retrospective nature of this analysis. This may also give way to under reporting of toxicity. Although each patient chart was reviewed using the CTCAE v4.0 reporting criteria for toxicity, lack of a central review or definitive protocol during treatment allowed for physician bias when symptoms were entered into the medical record. Size is another limitation of this study. Although this study evaluated 100 patients, having only six local failures limits the ability to study potential correlations between LC and other covariates such as various methods of tumor tracking.

## Conclusion

Stereotactic ablative body radiotherapy for the definitive treatment of early-stage inoperable NSCLC in the community cancer center setting has a LC and OS rate that is comparable to large academic institutions. Our risk adaptive approach of using the appropriate fractionated schedule based on tumor location and proximity to critical structures may explain for a very favorable toxicity profile. Future studies on CTC may identify patients with a high risk of distant progression and predict for the benefit of systemic therapy.

## Conflict of Interest Statement

Rachelle Lanciano, John Lamond, Stephen Arrigo, and Luther Brady possess partial ownership of Philadelphia Cyberknife. Alexandra Hanlon received a statistical consulting fee for analyzing data pertaining to the research. The remaining authors declare that the research was conducted in the absence of any commercial or financial relationships that could be construed as a potential conflict of interest.
